# Selections and Abstracts

**Published:** 1904-03

**Authors:** 


### SELECTIONS AND ABSTRACTS.

---

### Recent Developments of the Tuberculosis Problem.

If there is anything under the sun upon which is would seem difficult to say anything new, it is consumption. It has so long attracted the attention of the medical world, so many laboratories have been devoted to research work upon it, so many special societies have been organized to study it, so many organizations formed for the purpose of preventing and combating it, that it would seem as if the last word must have been said about it by this time.

It will be long, however, before this will happen. If for no other reason than that it continues to kill its one-seventh of all who die. And, apart from this great claim upon our attention, there is probably no disease of which our knowledge is expanding more rapidly and upon which our views are undergoing such marked and extraordinary changes. Twenty years ago it was supposed that the beginning of the last word had certainly been said by the discovery and identification of the bacillus. All was over except the application of the germicide. But the germicide has not yet been found, and its discovery appears to grow more remote and more improbable every day. In the meantime we are curing the disease by methods which utterly ignore both the germicide and the germ. Nor are our views as to the precise origin and method of spread of the disease in a much more settled or hopeful state. The discovery of the bacillus and its supposed identification with that of bovine and avian tuberculosis seemed to leave no room for doubt or question about the matter. One case was infected directly from another, or by the consumption of infected meat or milk; and to this day a majority of our profession are rather inclined to become indignant and show a tendency to hurl epithets, and other things that come handy, at the head of any one who is rash enough to dispute this dogma. But the last two years have changed all this.

First came Koch's bold statement that bovine tuberculosis could not be communicated to human beings, thus closing one wide source of infection. This position, of course, was not by any means universally adopted or accepted; quite the contrary. But it brought with it some unexpected and interesting results. In order to support it, he had to critically examine the evidence in alleged cases of infection which had hitherto been relied upon to prove the transmissibility. This was promptly done by Koch, Theobald Smith, Adami, Bovaird, and the writer, with the unanimous result that the evidence was found utterly inadequate either in amount or in character to make the possibility of transmission even reasonably probable. But, like the introduction of the higher criticism into certain regions of Christian theology, this method of challenge could not be restricted within the limits originally intended, but showed a distinct inclination to spread. It began to be applied to the evidence for the communication of tuberculosis from one human being to another; first by Adami, the writer, Ravenel, and finally by no less an authority than Behring himself. This last, although vigorously controverting Koch's position that human tuberculosis could not be communicated to bovines, and *vice versa*, goes on most unexpectedly to challenge the probability of human tuberculosis being transmitted from one case to another. His position is as striking and extreme as Koch's, for not only does he hold that bovine tuberculosis may be transmitted to human beings, but he goes on to declare that this is the *only* source through which the disease is acquired by our species. And while one can not say that either he or Koch have proven their positions, if for no other reason than that they are arguing by, so to speak, converse syllogisms; that is, that since human tuberculosis is or is not transmissible to cattle, therefore bovine tuberculosis is or is not transmissible to human beings; yet, Behring has certainly made out a very strong case against the usually almost axiomatically accepted belief that tuberlosis is actually transmited from one human being to another. He declares that nearly every one at some time during their infancy or childhood is invaded by tubercle bacillus, chiefly by the drinking of infected milk; that tubercle bacilli may remain dormant in the tissues for years. That when an individual develops tuberculosis this is chiefly due to the fact that the external conditions have become so unfavorable as to lower his general vitality and permit the dormant bacilli, lodged in some nook or another of his tissues, to take on new growth.

He regards the transmission of the disease to an adult as exceedingly improbable. This, of course, at first sight, looks like rank heresy, but we would earnestly adjure all who feel inclined to so pronounce it to just quietly devote a little time to a careful and dispassionate review of such evidence as is usually presented of the actual transmission of tuberculosis from one case to another. Unless his experience is exceedingly different from our own he will find that the confidence with which we have been accustomed to repose upon this belief is, to say the least of it, inadequately supported.

For instance, as regards the communicability of bovine tuberculosis, a careful search through the literature, both veterinary and human, by three different observers, resulted in the discovery of *only thirty-seven* cases of reasonably probable transmission through meat or milk in twenty years, and before the last of this list was published Koch had completely disproved the validity of five of these thirty-seven cases. This group of cases was such an interesting one, and has always been so unanimously placed at the head of the list by all believers in the transmissibility of bovine tuberculosis to human beings, that it is worth special mention. It was a group reported by the French physician Olivier, of five cases of tuberculosis occurring among the pupils in a certain boarding-school. No other case of tuberculosis could be traced in the families of any of the patients, nor had any other occurred in the older inmates of the school; but upon investigation of the cows from which the milk supply of the school had been obtained, one of them was found to be in a well-developed stage of tuberculosis, with involvement of the udder and bacilli in the milk. This was sufficient for Olivier, and the case was mooted abroad as an indisputable instance of direct infection. Several years afterwards Koch, in the course of his investigations referred to, took up this nstance, together with other alleged ones reported, and carefully investigated the facts personally, with the somewhat astounding result that he found conclusive evidence that *none of the milk of this diseased cow had been given to the pupils, but all had been consumed by the servants* of the school, not one of whom had developed tuberculosis.

This is a fair sample of what is meant by the loose and inadequate evidence of causation, which has been far too frequently accepted in the past.

As to the evidence for the direct communication from one human being to another, the bulk of the evidence is almost equally surprisingly small. The only large series that we have been able to find have been those of the now classic groups of cases of alleged transmission from husband to wife and wife to husband, collected by the Collective Investigation Committee of the British Medical Association in 1883, 158 instances; by the Berlin Medical Society, forty cases; and by the Medical Society of the Paris Hospitals, 107 instances—a total of 305 from France, England and Germany.

When we remember that these figures represent the total number of coincidences of this sort occurring in the experience of, at moderate estimates, from 500 to 1,000 physicians—the British list was based upon 267 affirmative replies—making less than one instance to each physician interrogated, and that nearly any physican can number from five to twenty-five patients who have lost a husband or wife by tuberculosis, the probable percentage of infection in such cases is not shown to be very large. Lists which do not give the total number of patients exposed to such contact-infection are of no value whatever as a basis for conclusions.

In the only series of cases discoverable in which this was done, that of Provost, the actual mortality by tubercle among the total number of survivors of such marriages was found to be no higher than that of the rest of the community. The whole question is in urgent need of further investigation upon broader lines.

As to the well-known and statistically abundantly proven fact that tuberculosis tends to "run in families," our knowledge is really on close scrutiny of the vaguest and most confused description.

The data so far collected are imperfect and incomplete. They are usually concerned solely with positive instances, or consist of a list of tuberculous individuals with the percentage of "family history" of the disease, without any statement as to the total size of the family groups under consideration in each case. When one in seven of the entire community die of the disease, it is obvious that any family group of seven or over is liable to contain one case of tuberculosis. Statements of total number of cases observed, or of descendants of tuberculous couples, are as urgently needed here as in conjugal infections.

This, of course, not to say for a moment that there are not innumerable instances of the occurrence of several cases of the disease in the same family, in the same house, or even in the same workshop or office. But that the possible sources of error in the parallel are so numerous and the number of other factors, which have been operating in this direction, such as identical food, identical sleeping-rooms, drainage of the house, ventilation, lighting, etc., to say nothing of the inheritance of similar defects of constitution, when the disease occurs in more than one member of the same family, is so great that it is almost impossible to find even a single case where one can show with a reasonable probability of certainty that the infection was directly transmitted from some other known cause. In short, Behring completely throws us back again upon that factor in the problem to which some of us have been urging the need of more careful attention, for several years past, namely, the soil in the place of the seed. He declares that the seed is present almost everywhere and it is simply a question of whether the conditions become favorable for its development as to whether the disease develops or not.

This, of course, is not new; it has been said scores of times before by pathologists like Virchow, and clinicians like Clifford Albutt. It has passed into a proverb in Germany, *Yeder Mann ist am Ende ein bischen tuberkulose.* ("Every one sooner or later has a touch of tuberculosis.")—*Medical Sentinel.*

---

**This Journal and Atlanta Semi-weekly Journal one year for $1.25 cash in advance. No checks. Strictly to new subscribers.**

### BURNS: PROGNOSIS AND TREATMENT.*

Modern methods of wound-dressing have caused the old classification of burns by Dupuytren to be somewhat revised, so far as prognosis is concerned at any rate; for prognosis is to be based upon the asepsis that can be secured and maintained.

Instead, then, of the six degrees of burns as formerly designated, we have practically but three:

1. An erythematous inflammation of the skin without vesication. This is due to the action of the heat producing an overfilling of the smaller arteries from a simple paresis of their constrictor muscles.

2. Those burns in which the inflammation of the skin results in the formation of vesicles; in these there is an exudation of serous fluid into the tissues, particularly the rete Malpighii; a portion of the epidermal layer is lifted up, constituting the blister.

3. Those in which there is partial or complete carbonization. Burns of the third degree are the result of albuminous coagulation, effecting the contents of the vessels, the serous fluid and albuminous substance of the tissues. Greater or less areas are deprived of nourishment and necrosis of tissue follows.

An exaggeration of this degree constitutes the fourth and fifth degrees mentioned by some authors, these terms being applied to either charring of the skin, or of the skin and muscular structure as well.

This includes those burns where sloughing forms are the secondary result of inflammation, and it covers the four more or less intense forms as classified by Dupuytren.

*Constitutional Symptoms*.—The period immediately following the injury through forty-eight hours is often one of great anxiety; pain is invariably present, varying from a slight degree through to most excruciating agony—congestion of surrounding parts or of internal organs, shock, collapse, convulsions, asphyxia and poisoning from absorption of products of burnt tissues.

Later, from the second to eighth day, there may be inflammation

---

*Read by Laura House Branson, B.S., M.D., Iowa City, Iowa, before the Johnson County Medical Society, Iowa City, Iowa, November, 1903, and the Iowa Union Medical Society Cedar Rapids, Iowa, December, 1903.

of part, with formation of slough—ulceration and hemorrhage may occur. Still later, there is separation of slough, followed by the process of resolution; there may be fever of a sthenic nature after the first twenty-four hours.

*Histology* shows that the skin is composed genetically of two main constituents, viz., an epithelial tissue, the epidermis, and a connective tissue, the cutis—each of these consisting of subdivisions. That the nerves of the skin are almost exclusively sensory and penetrate into the lower stratum of the epidermis, terminating either as free sensory endings or in tactile discs. Consequently, this explains the extreme pain experienced when the superficial portions of the body are burned, and it explains also death from shock in a burn of the second degree. The most painful burns, then, are those in which the outer layers of the epidermis are destroyed, leaving exposed these nerve endings. Death may occur in any one of these periods, the causes of death depending upon the extent of the injury, the depth of the burn and the part affected.

If death occurs early, it is due, usually, to cerebral coma or shock; over-stimulation of the superficial sensory nerves may cause cardiac paralysis.

Later, death may be the result of deep inflammation, intestinal ulceration, hemorrhage, uremia, pyemia, septicemia, tetanus or exhaustion from prolonged suppuration.

*Prognosis* depends upon the extent and depth of injury and also upon the part concerned; an extensive superficial burn is more dangerous than a deep one, limited in extent.

There are many proofs showing that mere reddening of two-thirds of the cutaneous surface will almost inevitably result in death, while destruction of one-third of the skin will probably produce the same result.

In burns of the third class the region affected, depth of tissue destroyed, age, sex, health, etc., will determine the result; slight burns of this degree may result fatally in youth and old age, as in childhood and in age the system generally suffers more severely from burns than in the adult.

Some physicians of known experience and knowledge claim the probability of perforation of the duodenum as one reason for a guarded prognosis. It is true, no doubt, that in this injury the intestinal as well as the respiratory tract is prone to congestion, followed by inflammation; this is true, also, of most of the internal organs, the explanation of these phenomena being that as one part of the physical economy loses its function, other parts have, in consequence, more work thrown upon them in order that nature's beautiful law of compensation may not be violated. Why the duodenum should be selected as the seat of greatest injury has not as yet been satisfactorily explained.

*Treatment.*—We find that the general education along the line of treatment in burns slight in nature conforms roughly to that followed by physicians—namely, emollient dressings with exclusion of air, although twice I have been called in to find that some well-meaning friend or neighbor had, while awaiting the physician's arrival, made matters far worse by applying kerosene and then excluding the air. Kerosene, though, finds its place in the old armamentarium to relieve pain, but its use is questionable.

Local treatment consists in soothing and protecting dressings, then in disturbing as little as possible, using aseptic dressings if parts have not been exposed to septic influence.

Antiseptic cleansing is necessary when clothing or other foreign material has remained for a time in contact with seat of injury. If vesicles have been ruptured or carbonization has taken place, asepsis must be secured and maintained if possible, as in these conditions more or less infection may follow. Among the antiseptics used are found boric, picric and carbolic acid, zinc oxid, bismuth subnitrate, bichlorid and iodoform.

Care should be taken in the use of antiseptics when the burn is one of great extent, as such a surface is readily absorbent, and the unlooked-for complication of drug-poisoning may arise, causing unfavorable results.

Dr. B. reports a case of burn of the second degree—himself the patient—in which iodoform was sprinkled freely over the surface; in due time symptoms of iodoform poisoning manifested themselves, to the surprise and chagrin of the attendant physician.

Among the emollient dressings our old-time friend Carron oil, now spoken of only in derision by some authors, still holds its own; linseed-oil varnish composed of lead oxid, 1 to linseed-oil 25; salicylic acid, 5 to 10 per cent., painted over part and covered with cotton-wool, is of known value.

Normal salt solution is proving itself to be an invaluable dressing.

Clay preparations, I believe, will in time take an important place in the treatment of burns, picric acid with clay being one of the newest remedies.

Baths may be used to facilitate separation of slough, but they should be discontinued as soon as they have served this purpose.

In tardy formation of granulations silver nitrate or balsam of Peru may be used, as in similar conditions from other causes.

*Constitutional Treatment* is of great importance; death may result at various periods and from different causes; consequently, our treatment must be modified accordingly. It consists in promptly meeting the symptoms at first and in forestalling them later on (shock and pain calling for urgent and heroic treatment) in regulation of nourishment, of exercise and of secretions, in counteracting the effects of suppuration and in treating any complication that may arise.

*Scars and Deformities.*—One of the greatest cares of the physician is the prevention of scars and deformities; he must be ready to meet the indications in each case, and by active and passive motion, by position, by splints and by skin-grafting, prevent or overcome.

Deformities as the result of burns are met with frequently, and time will not allow me to more than mention them and then to relegate them to the surgeon, to whom so much of credit is due for the restoration of the function of parts restrained by cicatricial contractions from this too common injury.

Here plastic surgery, so rapidly advancing, plays a great part, in that it virtually restores the dead to life by restoring to an organ or part its lost function.

Before leaving this part of the subject I want to call attention to a case recently reported by Dr. Roswell Park, in which an enormous epithelioma developed over the scapula in a cicatrix, resulting from a burn, and carcinomata resulting from cicatrices of burns—not so rare that we can afford to ignore them in our prognosis.—*Courier of Medicine.*

---

### THE PHYSIOLOGICAL ACTION OF DRUGS IN PATHOLOGICAL CONDITIONS.*

Perhaps a more proper heading for this paper would be: "A Plea for a More Scientific Treatise on Therapeutics."

No other element creates the criticism and distrust of the practitioners of the regular school of medicine as does their wide differences of opinion of the value of a given drug in a given case. It may be truthfully said of us that our ranks contain medical fanatics, medical skeptics, and a few, very few, medical scholars who place a rational faith, and no more than a rational faith, in the remedial power of any medicinal agent.

The fanatic is often youthful in years and far more theoretical than practical. With his hypodermic syringe in hand he wanders across field and brook, bagging the innocent animal of diminutive dimensions and administering to it inordinately large doses of his favorite drug. From these experiments he deducts his definite idea that this same drug will, without fail, produce like results in any case. The result is that he gives, and continues giving, dose after dose of his favorite nostrum, with the result that, instead of getting the physiological effect of which he is so sure, he gets a glimpse of the white horses, the hearse, and six gloved men. Our fanatic is now likely to join the ranks of the sewing-machine agent, horse-trader or a similar pursuit which permits of both more money and the exercise of a greater degree of veracity than does the practice of medicine.

And why this fatality? Why these disastrous results? Simply because our theorist was impractical enough to lose sight of the well-known fact that one should never whip a jaded horse. In other words, beware of administering a stimulant when the mortal mechanism demands the soothing effect of a sedative.

---

*Read by A. G. Emerson, M.D., Lewiston, Neb., before the Pawnee County Medical Society, December 7, 1903.

So much for our fanatic. He has simply learned the physiological action of drugs and attempted to gain their characteristic effect when the condition of his subject was not physiological but pathological instead. As intimated, he may have left our ranks for those less irksome and more lucrative, but the chances are that he is still with us in the role of medical skeptic.

Let us also analyze the character of this individual; for by this method I believe I can more deeply impress upon you the point I desire to make, namely, that our therapeutics are notoriously unscientific, which results disastrously to a unity of opinion. This is true because the field of therapy has been overlooked in the rush toward bacteriology, asepsis and surgery.

Our medical skeptic is, strangely enough, a man with a thriving practice, a level head, if you please, and generally a good, honest heart—because, if he were not honest, it would never be known that he was skeptical. He is thorough in dietetics, is rigidly aseptic, and generally very partial to hydrotherapy. He is master of psychology and has gained a grand reputation on diagnosis, and is almost perfect in point of prognosis. And why? Because his symptoms are not masked by medication, and the course of a disease is neither shortened nor made more severe by medicinal agents. In fact our skeptic would be an ideal man were he not narrow-minded enough to imagine that castor oil, calomel, sodium bicarbonate and whisky are not medicines.

You observe his inconsistency. He scoffs at drugs and yet unconsciously, you may say, he wields these great and powerful weapons, and goes on doing good by the very means he pretends to deplore.

Have you met our medical skeptic? Is my paper a farce, and too light reading for our Society, or is there an undercurrent of fact herein that should awaken an effort to discover the cause of an erroneous condition within our ranks?

And now, having advanced from the fanatic to the skeptic, it may seem strange that we have but a step farther to our ideal—the medical scholar. He it is who rivals the skeptic in popularity and excels him in the practice of scientific medicine. He, too, uses the few drugs employed by the skeptic; and he also uses many more, and uses them with a master hand, knowing exactly what to expect from the administration of his agent, because he weighs all of the indications carefully and then chooses the agent and the amount of the agent to suit that particular case.

The medical scholar knows that there is a difference between health and disease. He knows that the physiological action of a drug, in the strict meaning of that term, would be the effect obtained by administering that drug when the anatomy and bodily functions were normal. He knows that when an abnormal condition obtains the same dose of the same drug may cause widely different results. This point is really the theme of my paper. And what shall I write? My experience in the practice of medicine is of less than three years' duration, and that largely made up of mistakes. Our literature is very loose and unscientific on the subject. Three pages are devoted to the physiological action, and then a line added stating that the agent is often employed in pneumonia, nephritis, etc.

Now the field is open. The problem is unsolved. We have not gotten our agents to be used in a given case reduced to a system, and right there we are unscientific. Yet, one may say, you would have us become Hannibalites or Scudderists—they have it written in letters of gold, in major type, just what to administer as a specific. My answer is that Waugh and Abbot also specify, but, to my certain and sorrowful knowledge, their calcium sulphide does not always destroy pus.

It is not specifics that I would ask for, but rather a scientific explanation of how your specific gets in its miraculous action. I would plead for a materia medica that would begin with "A," and under "A" place Aconite, and after Aconite place Asthma, and they tell me how this agent acted in that particular pathological condition. And then pass down through all the list, ringing the changes of the action of the aconite in all the diseases, then carry all the agents through all the diseases and give us a scientific explanation of the action in each case.

One may say, what a waste of print and paper! And I reply: Why are your piles of rubbish on stomachs, and the kidneys, tumors and germs, so complex that the general practitioner can not afford time to search out this point, while the specialist is too envious to read his rivals' work? Whereas, a carefully indexed materia medica could be instantly instructive, and one need read but of the action of one drug in one case if he so desires, but that one would be found and its action recorded in every disease, from diabetes to a sour stomach.

Again, one may say, but you would never think of giving bromides in a condition of collapse from shock. Then, why waste time telling not to give them? And I would answer that I want to know why not. I want to have it tried, I want to be scientific, and in my mind we are desperately unscientific.

One authority advises vasomotor contractors in hemorrhage in order to close the bleeding ends of a vessel. Another says dilators, for the vasomotors check the flow by yielding less pressure to expel the blood. Both may be right, but science generally has but one right way—and I plead for a scientific materia medica.

We are too far apart—our ideas are too divergent. Let us urge on our pathologists and encourage our hospital experts to try out the different drugs in the various diseases and report the results. I predict that the time is not far distant when a series of surprises await us in the field of therapeutics. My belief is that the action of a drug in the same individual is as greatly modified by the different diseases as is the drop of water as it falls upon the bar of steel—to-day in the shade—to-morrow in the sun—again in the crimson forge, and at last on the snow-white drift where it instantly becomes a speck of ice. It was the same drop of water, the same bar of steel, but what of the results?

And so, to me, it seems that we have been following a false god. We administer that which acted well in health, but we forget that the frail system may be to-day in the shade, to-morrow in the sun, again in fever's forge, or upon the icy pinnacle of shock. In our most critical cases we choose our most powerful agent and seek to obtain the physiological action, but too often we gain terrible inaction instead.—*Western Medical Review.*

---

**This Journal and Atlanta Semi-weekly Journal one year for $1.25 cash in advance. No checks. Strictly to new subscribers.**

### TREATMENT OF CHRONIC BRIGHT'S DISEASE.*

The treatment of this pathological change in the minute anatomy of the kidney means: The protection of the affected organs and the maintenance of the body as a whole. For convenience we may divide it into Dietetic, Hydriatic and Medicinal.

The dietetic treatment of chronic Bright's disease should be based on certain principles as regards the final utilization of the foods in the organisms. One of the principles of dietetic treatment is the protection of the kidney-cells themselves against irritating by-products left over from certain classes of foods, bearing in mind that the organism as a whole must be nourished as well as the kidneys protected. Therefore, I am not an advocate of an exclusive milk diet in the treatment of chronic Bright's disease, but believe to get the best results we must supply a mixed diet whenever possible.

In a large number of cases of chronic Bright's disease we have a disturbed digestive system to deal with, and we must bear in mind these facts:

1. The chemistry of the stomach.
2. The physiological influence on secretion by the food itself.
3. The by-products which will be left over from each class of foods that will circulate through the kidneys. Examples of this group—urea and creatinin, both end products of nitrogenous foods, both active irritants and excitants to the epithelial cells of the kidney. Therefore, red meats should be limited in chronic Bright's disease.

Milk diet contains a large amount of phosphoric acid, which is an irritant also, and should be given in moderation. Lime water added to the milk reduces the per cent. of phosphoric acid by neutralizing it in the intestinal tract, and allowing the phosphates to escape in the feces. Water itself is a stimulant to the kidney and in the acute stage should be given in moderation. Proteids and fats in small amounts; proteids in the form of white meats, cereals and vegetables. Milk is a valuable food-product and should be

*Read by Simon P. Scherer, M. D., Indianapolis, Professor of Digestive Diseases and Physical Diagnosis, Central College of Physicians and Surgeons, before the Indianapolis Medical Society, November 24, 1903.

given in quantities not to exceed 45 oz. per diem. Cereal broths and broths of leguminous foods are valuable, as are fruit juices and a moderate amount of acetic and malic acid from fruits.

The intelligent prescribing of a diet in chronic Bright's disease is one of the most important parts of its management. The better the individual is able to digest and oxidize a mixed diet the better the prospects for his comfort and recovery. Warm clothing, avoidance of sudden atmospheric changes, cheerful surroundings and encouragement are all important factors in the management of diseases of the kidneys.

I am not an advocate of depleting these cases; the only times when such treatments are necessary or useful is when there is much edema, hydremia or threatened uremic coma. Many experiences and examinations have failed to prove that solids or toxic material in very large amounts can be eliminated through the skin; consequently the only hope from such treatment is the elimination of the excessive liquids. Long-continued depleting can only do harm as a routine treatment in this disease. When used at all it should be used with great caution, and its effects closely observed. The dry hot air, or the hot pack has been a method pursued successfully in the treatment of these conditions where depletion was required. Von Noorden highly recommends the electric-light bath, and I believe it to be an admirable method of diaphoresis. I have frequently had good results in threatened uremic convulsions by the hot pack, in addition to other methods of medication in such cases.

The use of drugs is one of moderate utility. The same rule applies here as in the dietetic and hydriatic treatment, viz., to maintain the integrity of the blood and to treat the accidents as they arise in the course of the disease, and to nourish the individual. Alcohol in general should be interdicted. Red wines and beer are allowable in very moderate quantities in some stages for their heat-producing value. Alkaline mineral water, as vichy and kissingen, with a little claret are allowable at dinner. Moderate use of tonics in the more chronic cases is of value. The old routine tonic treatment to my mind is not good practice. Organic iron, as a rule, accomplishes very little and may lock up the secretions and do positive harm. I see no good reason for giving quinine, as ordinarily it irritates the stomach and does more harm than good. Strychnia in moderate doses, not continued for more than a week at any period with an interval of a few days is good treatment. Hemoglobin et arsenic, or bovinine are both useful blood-making products. The old formula, Basham's mixture, in cases that are not secreting a large amount of urine, is useful and has served me a good purpose.

I have frequently observed in the treatment of chronic Bright's disease that the first thought of the attending physician has been to give diuretics. Here, as in the treatment of all forms of heart disease, where digitalis seems to be the first thought, potassium acetate, buchu, juniper, uva ursa, diuretin, and other active diuretics seem to be the first treatment. Early in the disease diuretics are not needed, as the kidneys need rest. Later in the disease they only act as a whip to goad the overworked cells, and only rarely do good. Late in the disease, with edema and hydremia, milder diuretics that act to increase the blood-pressure may be given. The natural aperient waters may be given and an occasional dose of blue mass to relieve the kidneys by more copious catharsis. Among the mineral waters that produce watery stools I find Freidichschalle water, and some of our own mineral waters from Indiana to serve a very good purpose.

Another product that I have used quite a little in the treatment of anasarca and edema associated with Bright's disease is five-drop doses in water of the specific tincture of oxydendron. I have had good results from this product. Very high arterial tension may be overcome by nitroglycerine.

All complications should be met and treated accordingly. For pleurisy—rest in bed, free catharsis, Dover's powders, counter-irritation of the affected pleura. The treatment of pneumonia should be the same as that disease alone, plus the management of the parenchymatous or interstitial nephritis which we have already instituted.

Dyspeptic symptoms require rest to a high degree; bland, unirritating foods in easily digestible forms.

Finally, in uremia, elimination should be the watchword by every means at our command. The hot pack, hot dry air, pilocarpine hypodermatically, chloral and chloroform should be exhibited in convulsive states. Blood-letting in full-blooded individuals is advisable. There is no specific medication, and here, as in other chronic conditions, good judgment, meeting the symptoms as they arise, should be the mode of procedure.—*Indiana Medical Journal.*

---

### THE CAUSATION AND TREATMENT OF ECLAMPSIA.*

In the April number of the *Annals* there is an excellent report of a most interesting meeting of the Obstetrical Society of Philadelphia, in which two papers by Dr. Charles S. Barnes and Wilmer Krusen on the "Etiology and Treatment of Eclampsia," respectively, were read, and which gave rise to a most instructive discussion. As no one, however, had a very positive opinion as to the cause of the condition, I would like to draw further attention to the theory which I advanced at a meeting of the Medico-Chirurgical Society of Montreal about a year ago, namely, that eclampsia is due to anemia of the corpora striata of the brain, or such other parts of the brain centers as control muscular movements. This anemia is brought about by a spasm of the arterioles which carry blood to the part; but the spasm is not limited to these arterioles alone; every capillary in the body is probably equally affected by the spasm, so that as the quantity of poisonous material, not necessarily urea, accumulated in the blood increases, the arterioles of the kidneys contract tighter and tighter until no more blood can get into them, and the spasm-exciting material rapidly increases in a vicious circle. As the spasm-producing toxines increase, the arterioles of the brain tighten up, causing disorders of vision and headache, and when the quantity of spasm-producing material has increased still more the circulation of blood through the kidneys comes to a stop, the circulation in the brain practically comes to a stop also, and the patient loses consciousness and unconscious or reflex movements called convulsions begin. The heart acts in correspondence with the vasomotor system, so

*By A. Lapthorn Smith, M.D., Consulting Gynecologist to the Women's Maternity Hospital, Montreal; professor of Gynecology in the University of Vermont, and of Clinical Gynecology in Bishop University, Montreal; Gynecologist to the Samaritan and Western General Hospital and to the Montreal Dispensary.

that the same poison which causes the spasm of the arterioles of the kidneys and of the brain also causes excited action of the heart, which is increased still further by the throwing of all the peripheral blood into the large veins and arteries, as is evidenced by the pulmonary obstruction. With this theory of spasm, and with it alone, can we answer any questions that may be put to us concerning the treatment.

What is the best treatment of eclampsia? One that will in the quickest possible manner and with the least possible danger put an end to that fearful vasomotor spasm and allow the blood to rush into the brain and kidneys so that the woman may regain consciousness, and so that her kidneys may at once begin to eliminate the toxin which causes the spasm. Within three minutes of seeing the case I administer a half or three-quarters of a grain of morphine. Is there any other drug which has a remarkable and almost specific power to relax the spasm without doing any harm? Yes; ten drops of tincture of veratrum viride hypodermically will relax that spasm in ten or fifteen minutes; we can not see the arterioles dilating under its influence, but we know that it is dilating them by our finger on the pulse; we feel the pulse coming down from 160 to 60 and even 40, growing softer as it slows. Whether the heart beats a hundred and sixty or only forty is not so important except as an indication that there is a let-up in the spasm of the whole vasomotor system, which is a matter of life and death; in the three cases in which I have used it there was no convulsion later than fifteen minutes after the first injection. When I returned from the meeting of the American Gynecological Society, where Dr. Reamy first advocated this remedy, I requested one of my pupils, Dr. DeCotret, who had just been appointed director of the largest lying-in hospital in Canada, to try this remedy, and so far he has employed it in over forty cases without a death; no case of eclampsia has died in the institution since the veratrum treatment was instituted. Such being the case, I feel it a duty to try and induce others who have many cases every year, and of which a large proportion die, to adopt the treatment I have indicated, namely, a hypodermic of morphine, followed in five minutes by a hypodermic of ten minims of tincture of veratrum viride, repeated every ten minutes until the convulsions stop or the pulse comes down to forty. Then give a quart of salt solution by enema; it will be quickly absorbed by the rectum, and as the arterioles of the kidneys relax the water will rush into them, and in half an hour there will be a copious secretion of poison-laden urine. No chloroform will be needed, and no chloral, which latter drug has caused so many deaths ascribed to other causes; no accouchement force; just wait until the woman falls into a quiet slumber, half an hour in my three cases, and go home. There will be a normal labor and probably a dead fetus in a day or a week. You may get a fright when the pulse comes down to forty, but there is nothing to fear; I have not been able to find or hear of a case of eclampsia treated with morphia and veratrum but without chloral or chloroform that died. I firmly believe that the administration of a pound of chloroform in twenty-four or forty-eight hours, at the end of which time the woman died, is quite enough to cause her death without eclampsia. In the discussion at Philadelphia above referred to, every one who had used veratrum spoke well of it; why should not every one use a remedy which has given such wonderful results? It was the witnessing of the immediate benefits following its administration which led me to evolve my theory of spasm of the vasomotor system which has been favorably received by at least one physiologist of world-wide reputation. My article was published in a small local journal, and was, therefore, for the time being buried; but I trust that its wide publicity in the *Annals* will lead to the general adoption of the spasm theory and of the life-saving veratrum viride treatment.—*Annals of Gynecology and Pediatry.*

---

### ADVANCES IN THE TREATMENT OF PARALYTIC DEFORMITIES.

A. H. Tubby (*The Lancet,* Vol. I, No. 13, 1903,) gives the following varieties of paralytic deformities: (1) Those arising from anterior poliomyelitis; (2) those due to infantile spastic paralysis, infantile hemiplegia, and cerebral diplegia; and (3) those due to injuries of the nerves, such as the Erb-Duchenne type of palsy, affecting the upper root of the brachial plexus, or lesions arising from crushing or severance of nerve trunks. He dwells on the remarkable selective tendency for certain groups of muscles, individual muscles, or even part of a muscle, in a certain number of cases of anterio-poliomyelitis; also the fact that in any given case it is impossible to foretell the extent to which recovery will take place; and one is continually surprised from time to time by seeing an apparently hopeless limb resume under treatment all, or nearly all, its functions. The operation of the form of tendon-grafting to be preferred originated with Nicoladoni in 1882. A large number of cases are recorded. Vulpius gives his experience in sixty cases. The rationale of the operation is, first of all, to utilize what is ill-directed voluntary movement, and, secondly, to restore the balance of muscle so far as possible in the affected part. But it will be evident that the joint can never be a strong one, since the amount of power at the disposal of the surgeon has been diminished by disease.

*Various Methods of Tendon Transplantation.*—These are numerous and are constantly added to by surgeons. The principal methods used are represented in the article by a diagram from the article of Vulpius. In instances where the tendon will not reach to the point of its intended insertion, a bridge of silk or gut can be used with apparently fair success. Jochner has succeeded in bridging a gap of two and one-half inches in the extensor communis digitorum by a similar method. The author is probably right in thinking that cerebration, or its probable absence, at least for a period, plays any part in an improved state of affairs after the operation, for cerebration and muscle-balance seem to adapt themselves quickly to the changed conditions. It may be of interest to review briefly the most common pathological conditions due to the paralysis of certain muscles and means the author advises for their correction.

Talipes calcaneo-valgus can be treated by the insertion of the peroneus longus into the inner side of the tendo Achillis.

Talipes calcaneo-varus is treated by the insertion of the flexor longus digitorum into the outer side of the tendo Achillis, uniting the distal part of the cut flexor longus digitorum with the flexor pollicis.

Talipes equino-valgus is treated by splitting the tendo Achillis and the gastrocnemius up as far as the junction of the two heads of the latter and then inserting the inner portion of muscle and tendon into the tribialis posticus, or, better still, bringing it well forward and fixing it on the under aspect of the scaphoid bone. This relieves the valgus portion of the deformity. The equinus portion is readily rectified by section of the remaining half of the tendon of Achilles.

Talipes valgus may be treated by transplanting the proximal part of the peroneus brevis into the tibialis anticus above the ankle joint, and, if necessary, tenotomizing the peroneus longus.

In extensor curis paralysis the sartorius can be divided just above its insertion and inserted into the top of the patella. If the sartorius is defective, the semimembranosus can be taken from the inner side of the joint and a portion of the biceps from the outer side of joint and both inserted into the top of the patella.

Paralytic conditions of the wrist lend themselves especially well to tendon-muscle transplantation, but the author's remarks on this subject are not included in this review.—*Maryland Medical Journal.*

---

### CONCORDIA DISCORS—DISCORDANT HARMONY.

Members of the legal profession are entitled to the bar of another State through an introduction to the presiding judge, who then and there passes upon their credentials.

A commissioned surgeon or a physician of the federal government is in professional standing anywhere, at home or beyond the seas, and entitled to practice under the laws of the United States. The civil practitioner, who is also presumed to be a citizen of the United States, is not always so privileged. But there must be a reason for this.

"Full faith and credit shall be given in each State to the public acts, records and judicial proceedings of every other State." (Const., Art. iv, Sec. 1, Par. 1.) The State laws regulating the practice of medicine are so different in the different States, that in many, the privilege of practicing to one changing his State would be denied without retrograding to the status of an undergradute and passing an examination to qualify.

The details of anatomy, physiology and chemistry, the data of forensic medicine, toxicology and laboratory research, the minutiæ of capital surgery, etc., are subjects for reference, to prepare for which, the practitioner accumulates [a library and indexes the tomes on his shelves for consultation. No practitioner carries his talents at his wit's end in dealing with human life, but prepares his diagnosis and treatment as a weighty opinion, as the details of any momentous action are formulated by logical method and research.

"The citizens of each State shall be entitled to all privileges and immunities of citizens of the several States." (Const. Art. iv, Sec. 1, Par. 2.) Now it is fair to say, that if an authorized doctor of medicine going to another State with a diploma from a recognized college should register at the county or parish court, he would fulfill every requirement of the law. With such credentials, the authorities would be unconstitutional in refusing and liable, it seems. One impediment is the inability of the county clerk to recognize a good from a bogus diploma, which thus makes it often necessary to refer the matter to the State board of health. Constitutionally there is no provision made for this exchange. "No State shall enter into an agreement or compact with another State." (Const., Art. 1, Sec. 10, Par. 2.)

Why are the laws conferring upon medical colleges the right to issue diplomas placed within the jurisdiction of the police powers of the State? No health board is constituted a police board to discredit the qualification of the college diploma. The State of Kentucky authorizes one officer as a police magistrate only.

The remedy, to the writer, appears to be possible in one of two measures, viz.:

By common consent, of an agreement effecting the system of exchange through the channels of the State's board of health of State certificates. This might be secured by the joint action of the State's medical society.

Another plan, the fond dream of the civil practitioner, is the degree M. D. U. S.

This could probably only be attained by an amendment of the constitution of the various States, by adding thereto a section accepting a license to be conferred by a national board of examiners of health, the same to be constitutional authority in any of the States, Territories or insular possessions of the Union. This possibly would fall within the jurisdiction of the American congress of medicine in joint action with the national board of examiners, representing a conference of States. And Congress may, by general laws, prescribe the manner in which such acts, records and proceeding shall be proved, and the effect thereof. (Const., Art. iv, Sec. 1, Par. 1.) The subject has been canvassed and exploited and has suffered always defeat; it has been laid upon the table awaiting some more convenient time and different method of organized procedure.—F. SILSBY TRIPP, M. D., Pleasant Hill, Ky., in *Wisconsin Medical Recorder.*

---

### SUPPURATING APPENDICITIS OPENING INTO THE BLADDER.

BY DR. ENRIQUE FORTUN, SURGEON OF HOSPITAL NO. 1, HAVANA.

Juan G., a Spanish merchant, thirty-seven years old, with evident syphilitic antecedents, began to suffer about two months ago acute pains in the right iliac pit, while a tumefaction was observed in that region.

He became an inmate of a clinic of this city, where his case was diagnosed as malignant neoplasm. After remaining about twenty days in said clinic, the patient decided to leave for Spain ; in the meantime he stopped at a hotel here. While there he was taken with violent fever and ague, with a temperature of about 41 degrees C., and the first micturition following this attack did show the presence of a great quantity of pus.

Dr. Parra, who was attending the patient, did me the honor to ask me to assist him. I called on him the night after the evacuation of pus had occurred.

The first symptom to which my attention was called upon examination was the dimension and hardness of the liver, with swellings the massiveness of which continued uninterruptedly in connection with the massiveness of the iliac pit, in which region (the right iliac pit) an accentuated muscular resistance was observed, though that region instead of being swollen presented a depression, at the bottom of which the rim of the hepatic gland could be felt by the hand. The temperature was 38 degrees, the pulse beat between 80 and 90, and the general condition of the patient was rather satisfactory.

The diagnosis offered no doubt in our opinion: Suppurating appendicitis with evacuation into the bladder (the urine which was shown to us was extremely fetid and mingled, and it did contain a large quantity of pus) and syphilitic cirrhosis of the liver.

We advised the patient to consent to be operated upon, which he did. On the following day an incision of about 7 centimetres was made into the middle of the depression observed in the iliac pit. We rapidly reached a perfectly defined cavity, which contained a little pus mixed with mucosities. We washed out the cavity with *Hydrozone* and plugged it with iodoform gauze. On the following day, when we dressed the wound, upon careful examination of the cavity, we did not find any connection with the bladder, but we could extract the appendix which was effected by feces.

A complete cure was accomplished in a month, and during that time the liver decreased considerably in volume. Since the third day of the operation antisyphilitic treatment was followed.

The communication between the cavity of the abscess and the bladder healed after twelve days of treatment.—*Revista Medica Cubana,* of July, 1903.

---

### SURGICAL HINTS.

In hemorrhage from the umbilicus in infants, if pressure fails to stop it, pass two pins under the navel, at right angles to each other and apply a suture twisted below them.

When an artery of fair size has been cut through and, after tying the proximal end the distal end can not be found, it is advisable to pack the wound with gauze for twenty-four to forty-eight hours before finally closing it.

In order to be able to draw on rubber gloves easily, it is a good plan to place them in a lysol or creolin solution after they have been boiled. The soapy nature of these solutions lubricates the glove and hand.

In the treatment of fractures occurring in alcoholic subjects, remember that they are particularly liable to the development of delirium tremens, and that unless they are well secured in bed they may get up and attempt to use the injured limb with disastrous results.

In compound fracture with much splintering of bone, it is often inadvisable to operate extensively for the removal of the loose pieces. When the wound itself is small and uninfected it is often a risky procedure to enlarge it, and military surgery has shown us that loose pieces often do no harm and are either absorbed or reunited to the bone.

Never omit the ordinary aseptic and antiseptic precautions because a wound is already infected, or because you are operating in an open cavity. In the first instance you may bring about a mixed infection, and in the second it is well known that the open cavities can take care of a moderate number of bacteria only, and that the cleaner they are kept the better they will do it.

Study the pulse of your patients before operating and give the benefit of your knowledge to the anesthetist. It often happens that the excited and forcible pulse of a patient at the beginning of anesthesia returns to the weak and rapid state which existed before the operation, as soon as the anesthetic has taken full effect. This may lead to unnecessary and even dangerous attempts to stimulate a heart that is doing as well as could be expected.—*Inter. Journal of Surgery.*

---

### THE IODINE TREATMENT OF PUERPERAL SEPSIS.

W. R. Pryor contributes to the *N. Y. Medical Journal,* August 22, 1903, the result of his experience of septic cases treated by opening the vaginal vault and the introduction of iodoform gauze into the pelvis.

In every case of puerperal streptococcus endometritis, streptococci were found free in the pelvis, and in over 97 per cent. of cases they are present in the uterine contents.

After cleansing out the uterine cavity, he opens the posterior cul de sac and packs both with large quantities of iodoform gauze. In nearly all cases, after this procedure, cocci will be absent after the third dressing. The iodoform acts by causing local iodism and it is this that sterilizes the pelvis. Pryor reports thirty-seven cases treated by this method, twenty-seven of which had had no previous operation and only one died, while ten had been previously curetted by others and three died.

In all of the cases enteroclysis, or intravenous infusion accompanied the operation to aid in eliminating the iodin and toxins by the damaged kidneys.

In a later article (*N. Y. Med. Journal and Phil. Med. Journal,* January, 1904), in commenting on the morbidity of the cases treated by this method, in his own work and by others, Pryor says:

"We have found that six of the subjects have subsequently conceived, five going to full term, one inducing abortion at the fifth month. Contrast this treatment with that of curetting alone with a mortality of 22 per cent.; antistreptococcus serum, mortality of 33 per cent.; hysterectomy, mortality 55 per cent., and the let-alone treatment, mortality of 7 to 25 per cent. and we have nothing for which to apologize.

"But there is another phase to the subject. All of the women we have operated on and who have lived have kept their uteri, and six that we know of have had a restoration to physiological function."

### THE MANAGEMENT OF DISTURBED LACTATION.*

The importance of maternal nursing is insisted on for the reason that it increases the mother's affection for her offspring and gives the child a good start in life. In case a defect in the quality or quantity of the milk is present, as evidenced by the child not gaining in weight as it ought to, a chemical and microscopical examination will usually reveal the source of the trouble. The author's conclusions are as follows:

1. That the various medicinal and much-advertised galactagogues have been of little value.

*Jerome Walker, M.D., Brooklyn Medical Journal, January, 1904.

2. That cow's milk taken by the nursing woman in such quantity as can be digested improves the quality and quantity of the woman's milk better than gruels, panadas, soups and porridge. A mixture of one teaspoonful of tincture of ginger with one tumblerful of slightly sweetened milk is valuable, especially taken at night.

3. That an abundance of plain, wholesome food and regular action of the bowels are important. On the contrary, gross eating and poor defecation not infrequently lessen the quantity of mother's milk.

4. That regularity in times of nursing is most important, and also allowing the child to take all it will at each nursing.

5. That every day exercise by both mother and child in the open air, but not to the point of tiring, helps the child's digestive powers and increases the supply of milk. Exercise indoors even with the windows open, will not take the place of outdoor exercise.

6. That a cheerful disposition goes far towards keeping the milk in good condition. Anger, worry, excitement and insufficient sleep have the opposite effect. Some women, therefore, will never be good nurses.

7. That every nursing woman should wear easy-fitting, comfortable clothing, especially about the chest, abdomen and neck, and should keep heavy distended breasts from sagging.

8. That carefully selected tonics, laxatives and even hypnotics are at times necessary.—*St. Paul Medical Journal.*

---

### SYPHILIS AND TABES.

Dr. Erb, of Heidelberg, in a very lengthy and erudite article on this subject, one of a series, appearing in the *Berliner Klinishe Wochenschrift,* January 18, 1904, maintains that tabetic individuals exhibit in about 80 per cent. of cases a history of pre-existing lues and gives statistics confirming the assertion. He also points out that tabes may be the result of the hereditary form of syphilis, as evidenced by the cases occurring in his practice and which he styles infantile and juvenile tabes, according to the respective age of the patients. Of special interest are the "tabetic couples," cases where both husband and wife are simultaneously the victims of tabes dorsalis. Experience teaches that they were, without exception, at one time sufferers from syphilis. Such cases are not at all infrequent in medical literature. He cites the following very interesting observations: Boy, age five, contracted syphilis from his bedmate; at the age of eighteen he developed locomotor ataxia. The boy eventually infected his mother, and she in turn gave the virus luetious to her husband; both of whom are at present suffering from incipient tabes. (The above cases are typical cases of what is termed "syphilis insontium.") Cases of syphilitic tabes, paralysis, spinal myosis and other syphilogenic maladies of the central nervous system suggest the probability of the existence of a syphilitic toxine affecting the nervous system and giving rise to the various morbidities above enumerated. Pathologic anatomy concedes to syphilis the instrumentality in the production of gray degeneration and primary atrophy of the nerve-paths, manifestations similar to those observed in posterior sclerosis. Moreover, syphilomata have been known to occur concomitantly with tabes. A number of additional observations are detailed in this interesting essay, all of which go to prove the syphilitic basis of tabes dorsalis.—*Central States Medical Magazine.*

---

### THE PATHOLOGY AND SURGICAL TREATMENT OF BRIGHT'S DISEASE.

S. C. Gordon (*Annals of Gynecology and Pediatry*, November, 1903,) says that Bright's disease is primarily an acute inflammation of the kidney structure, not dependent upon any specific infective germ, which if early recognized and properly treated will terminate by resolution. If neglected or not recognized, repeated acute attacks may occur, each one leaving products of the inflammatory process, which may organize or even suppurate. Acute attacks are always short, but leave more or less products, which interfere with the circulation, finally producing a chronic passive congestion incorrectly called "chronic inflammation." The result of these attacks is an enlargement of the organs, causing pressure of the fibrous capsule. Complete decapsulation relieves this pressure, depletes the distended vessels by more or less bleeding, and allows the circulation to resume its normal condition and absorb the exudate. Even one kidney alone may be involved, and the symptom be relieved by operation upon that one only. The surgeon may be justified in operating even in cases far advanced when the suffering is great, simply for relief of the suffering.—*Medical Age.*

---

### NOTIFICATION OF TUBERCULOSIS.

Dr. William Osler remarked before the joint meeting of the Maryland Public Health Association, the Medical and Chirurgical Faculty and the Læennec Society, as follows, concerning his ideas of the treatment of pulmonary tuberculosis by the civil authorities:

Now, what is the remedy? It is simple. It is very easy. It is so simple and easy that we won't get it for a good many years. It is a sad thing to think of, but it will be five years yet before we get a law compelling notification of cases of tuberculosis to the Health Board of Baltimore. Yet we all know there should be notification and careful inspection in every single case. It works no hardship to have the Board of Health know that a case exists in the family, even in the best house at Mount Vernon place. It can be done quietly and easily, without placarding or making the patient feel that he is a social outcast. It can be done properly and easily under systematic management.

*The Medical Record* has always staunchly championed the cause of the consumptive, and of the needy consumptive in particular.

The notification of pulmonary tuberculosis—at any rate among the poorer classes, who live in close contiguity and are thus peculiarly subjected to the influences of the microbe—is a measure to be taken in the interest of public safety.

Sanatoriums for the treatment of consumptives alone have now decisively proved their worth, and the erection of such public institutions in the vicinity of every large city is an act of self-preservation on the part of its inhabitants.

